# A machine learning based delta-radiomics process for early prediction of treatment response of pancreatic cancer

**DOI:** 10.1038/s41698-019-0096-z

**Published:** 2019-10-04

**Authors:** Haidy Nasief, Cheng Zheng, Diane Schott, William Hall, Susan Tsai, Beth Erickson, X. Allen Li

**Affiliations:** 1 Department of Radiation Oncology, Medical College of Wisconsin, Milwaukee, WI USA; 2 Department of Biostatistics, University of Wisconsin-Milwaukee, Joseph. J. Zilber School of Public Health, Milwaukee, WI USA; 3 Department of Surgical Oncology, Medical College of Wisconsin, Milwaukee, WI USA

**Keywords:** Cancer imaging, Mathematics and computing, Cancer models, Prognostic markers, Tumour biomarkers

## Abstract

Changes of radiomic features over time in longitudinal images, delta radiomics, can potentially be used as a biomarker to predict treatment response. This study aims to develop a delta-radiomic process based on machine learning by (1) acquiring and registering longitudinal images, (2) segmenting and populating regions of interest (ROIs), (3) extracting radiomic features and calculating their changes (delta-radiomic features, DRFs), (4) reducing feature space and determining candidate DRFs showing treatment-induced changes, and (5) creating outcome prediction models using machine learning. This process was demonstrated by retrospectively analyzing daily non-contrast CTs acquired during routine CT-guided-chemoradiation therapy for 90 pancreatic cancer patients. A total of 2520 CT sets (28-daily-fractions-per-patient) along with their pathological response were analyzed. Over 1300 radiomic features were extracted from the segmented ROIs. Highly correlated DRFs were ruled out using Spearman correlations. Correlation between the selected DRFs and pathological response was established using linear-regression-models. *T* test and linear-mixed-effects-models were used to determine which DRFs changed significantly compared with first fraction. A Bayesian-regularization-neural-network was used to build a response prediction model. The model was trained using 50 patients and leave-one-out-cross-validation. Performance was judged using the area-under-ROC-curve. External independent validation was done using data from the remaining 40 patients. The results show that 13 DRFs passed the tests and demonstrated significant changes following 2–4 weeks of treatment. The best performing combination differentiating good versus bad responders (CV-AUC = 0.94) was obtained using normalized-entropy-to-standard-deviation-difference-(NESTD), kurtosis, and coarseness. With further studies using larger data sets, delta radiomics may develop into a biomarker for early prediction of treatment response.

## Introduction

Pancreatic cancer (PC) is a devastating malignancy and one of the leading causes of cancer deaths in the United States.^1^ Despite aggressive combined modality treatment approaches, the overall 5-year survival rate remains <5%.^2,3^ Local recurrence following definitive therapy remains a common and morbid event that occurs in 20–60% of all patients.^1,3–5^ Approximately 40% of PC patients present with locally advanced unresectable disease.^6^ A subgroup of these patients who do not develop metastatic disease may be cured with advanced treatment such as adaptive radiation therapy (RT) with high-radiation doses.^3^ Detecting treatment response in an early stage during the treatment is desirable to allow adjustment of the remaining treatment according to patient or tumor-specific response, and hence, delivering the most effective adaptive treatment.

Medical imaging is routinely used to monitor and/or predict treatment response for cancer treatment.^7–13^ Radiomics translates medical images into the quantitative data. It has been reported that image-derived radiomic features can measure spatial heterogeneity of a tumor and can detect spatial response variations.^14–17^ CT-derived textures have shown promising prognostic value in a variety of applications. For instance, Hou et al. performed radiomic analysis using contrast-enhanced CT and found that the identified radiomic features have the potential to predict treatment response in esophageal carcinoma (AUC = 0.97).^12^ Coroller et al. found that CT-based radiomic features can capture detailed information about tumor phenotype and can be developed as a prognostic biomarker to predict distant metastasis in lung adenocarcinoma.^13^ Radiomics has the potential to identify imaging biomarkers for the management of PC. For instance, Eilaghi et al. reported that CT texture features of the dissimilarity and normalized inverse difference were associated with overall survival for PC.^18^ Chen et al. showed that the first-order radiomic features, e.g., mean, skewness, and kurtosis, demonstrated significant changes during chemotherapy-RT (CRT) that can be correlated with pathological responses.^19^

Delta radiomics can assess the relative net change of radiomic features in longitudinal images,^17,20^ which can offer abundant information to identify, quantify, and potentially predict therapy-induced changes over the course of treatment. Delta-radiomics features (DRFs) can be derived from a variety of metrics in conjunction with clinical outcome. The presence of a trend in DRF during treatment may indicate a good or poor response to treatment. For instance, Al-Kadi and Watson showed that fractal texture changes in time-sequenced contrast-enhanced CT images could be used to differentiate between aggressive and nonaggressive malignant tumors with 83% accuracy and could potentially impact the clinical decision for choosing the appropriate treatment plan.^21^ Fave et al. used DRFs to create a model for survival and distant metastases finding that radiomic features such as compactness and texture strength improved the prognostic power of the model.^22^ However, as delta radiomics is in its infancy, a general methodology on how to identify DRFs is desirable. Thus, in this study, we developed a delta-radiomics process based on machine learning and tested the process by analyzing the longitudinal CTs acquired during CRT for pancreatic cancer.

## Results

The proposed delta-radiomics process and the machine-learning algorithms were successfully used to perform the delta-radiomics analysis on the selected patient data. Major findings are described below.

### Selection of delta-radiomics features

**Fig. 1:**
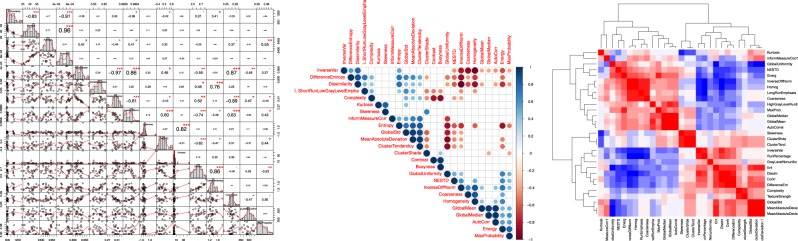
DRFs’ Spearman correlations. A sample of the correlation histogram with *p*-values is shown on the left, Spearman ranking (middle) for some of the DRFs, and an example of a Spearman Correlation heatmap for some of the DRFs (right)

It was found that based on the Spearman correlation coefficients obtained, a total of 73 DRFs extracted from the segmented pancreas head of each daily CT set had *r*_s_ < 0.9, i.e., not containing redundant information, thus, justifying the use of these features for the delta-radiomics analysis. This DRF set was examined across all patients to ensure its reproducibility. Figure 1 shows a sample of the numerical correlation coefficients, the histograms, and the scatter plot (left), Spearman ranking (middle) showing colored dots whose size and color correspond to the correlation value and white blocks representing insignificant coefficients, and an example of a Spearman correlation heatmap for some of the DRFs (right).

### Effects of respiration motion and CT acquisition

**Fig. 2:**
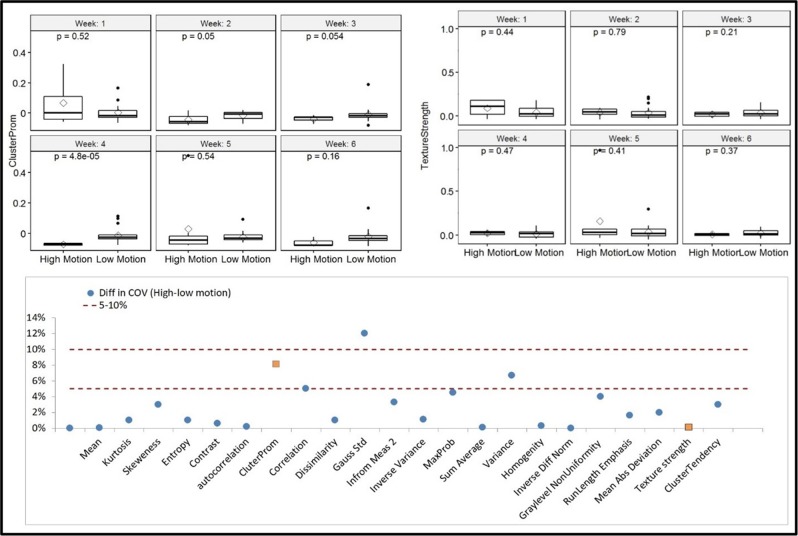
DRFs as a function of motion artifact. A comparison of two DRFs, cluster prominence (left), and texture strength (right), along with the *t* test *p*-value in different weeks during CRT between the low and high motion groups, and the difference in coefficient of variance (COV) between high and low motion for selected DRFs (right) with 5–10% levels indicated with cluster prominence and texture strength highlighted

To select the DRFs that are not substantially affected by respiratory motion, all extracted features were compared between the low and high motion groups. It was found that, among the extracted DRFs, 35% were affected by the motions with COV > 5%, of which 45% had a COV < 10%. The effect of motion was more apparent for the higher order features extracted on the daily CTs acquired between weeks 2 and 4 of the treatment. The mean, mean absolute deviation, autocorrelation, texture strength, inverse difference normalized, information measure, inverse variance, sum average, gray level nonuniformity, and the newly developed NESTD are examples of the features with less motion dependence (COV <5% and nonsignificant *p*-values from both the *t* test and the MSLR test). A comparison of two DRFs, one with large motion effect (cluster prominence, top left) and another with small motion effect (texture strength, top right) in different weeks between the low and high motion groups and the difference in the COV for selected DRFs (bottom) between the two groups are shown in Fig. 2. The weekly DRF value (top left and right panels in Fig. 2) combines the DRF values of all five fractions in the week.

For the effect of the different acquisition parameters (scanners), the *t* test and regression analysis showed that there was no significant difference (*p* > 0.05) in the selected DRFs between the two different acquisition protocols, indicating the DRFs can be used for all the selected patient data obtained from the two CT scanners.

### Correlation with treatment outcomes

It was found from the linear regression analysis that 27 out of the 47 DRFs that were not affected by both respiratory motion and image acquisition exhibited a trend to correlate with the pathological response. These DRFs include autocorrelation, cluster shade, cluster tendency, coarseness, complexity, contrast, difference entropy, dissimilarity, energy, entropy, mean, median, standard deviation, global uniformity, gray level nonuniformity, high gray level run emphasis, homogeneity, information measure, inverse difference normalized (IDN), inverse variance, kurtosis, skewness, NESTD, short-run emphasis, max probability, mean absolute deviation, and run percentage. The values of these DRFs were different between the good- and bad-response groups, indicating their associations with the responses.

**Fig. 3:**
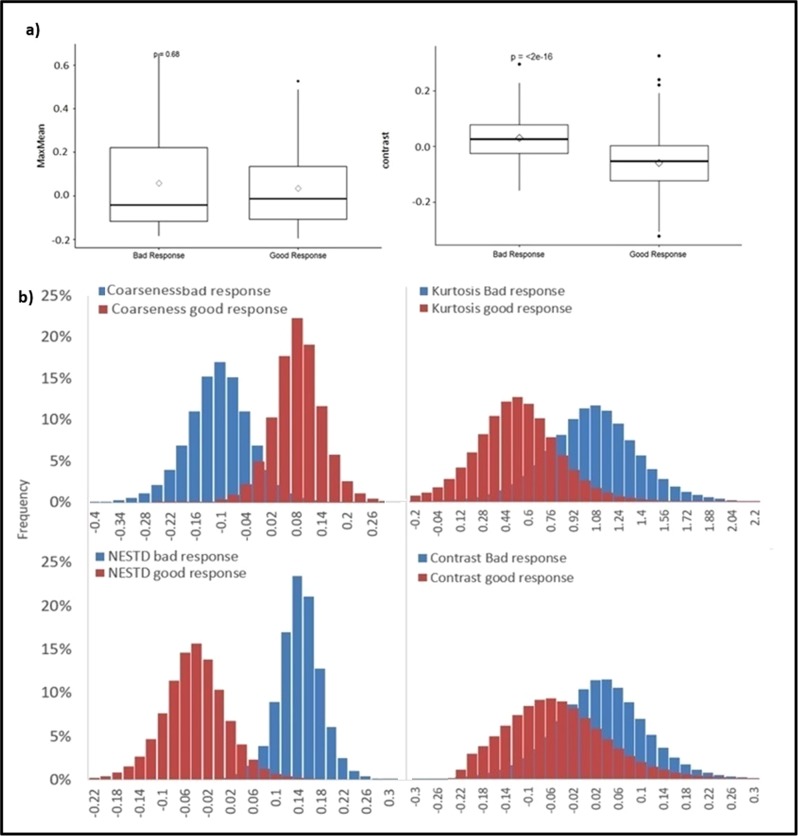
DRFs *t* test and distributions. Boxplots and corresponding *t* test *p*-value for (**a**) average DRFs of all fractions for maximum to mean ratio (MaxMean) feature indicating no significant difference between the two response groups and for the contrast feature showing significant differences. The presented boxplots show the median and interquartile range for each response group, and the diamond data point in the middle represents the mean of the group, (**b**) distribution of good- and bad-response group including all patients and all fractions within each response group for Coarseness, Kurtosis, NESTD, and contrast

Figure 3 presents comparisons of sample DRFs between the good and bad-response groups in boxplots, showing (a) the average DRFs for all fractions for the maximum to mean ratio (MaxMean, left) feature with no significant difference (*t* test *p-*value > 0.05) between the two response groups, where the DRF value for each patient was the average of all fractions, and the average DRFs for all fractions of the contrast feature (right) exhibiting significant changes (*p* < 0.05) between the good- and bad-response groups, the presented boxplots show the median and interquartile range for each response group and the diamond data point in the middle represents the mean of the group, and (b) examples of distribution of good- and bad-response group including all patients and all fractions within each response group for coarseness, kurtosis, NESTD, and contrast during the entire course of treatment.

**Fig. 4:**
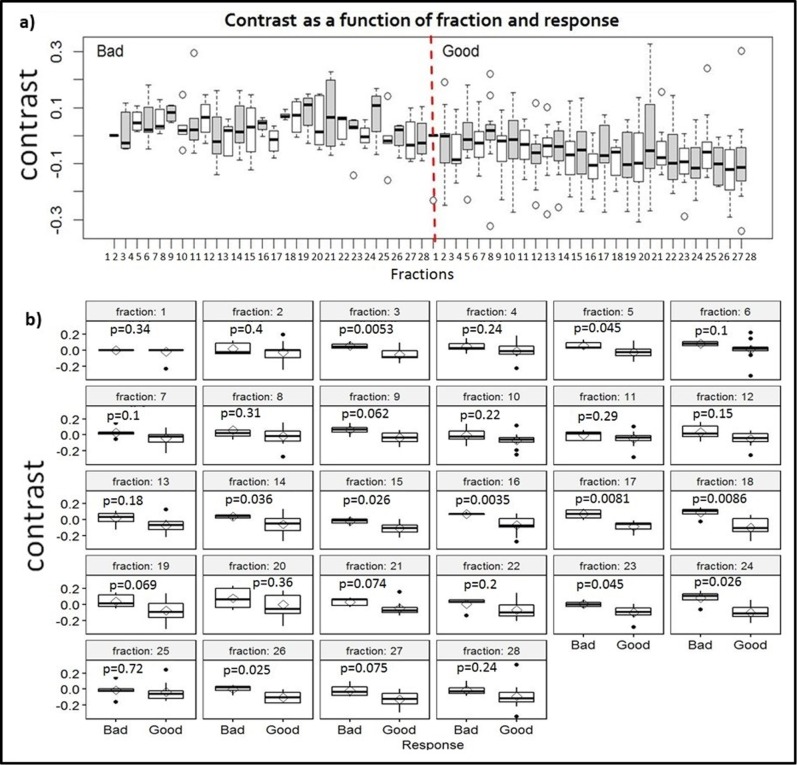
Fractional changes of the contrast feature. Comparisons of the boxplots and *p*-values of daily DRFs of the contrast feature for all patients in a response group between the two response groups, showing (**a**) all daily DRFs for both groups plotted together and (**b**) daily DRFs for the two response groups plotted for each fraction

Figure 4 compares the boxplots and the *p*-values of daily DRFs of contrast feature for all patients in a response group between the two response groups, showing (a) all daily DRFs for both groups plotted together and (b) DRFs for the two response groups plotted for each fraction. In fractions 14–18, the differences in the daily DRFs between the two response groups are significant in four consecutive fractions (*p* < 0.05).

Based on the analysis with *t* test and linear mixed-effects model, it was found that 13 of the 27 selected DRFs passed both tests with *p-*value < 0.05, indicating their significant correlations with the pathological response. These candidate DRFs included complexity, cluster tendency, coarseness, information measure, contrast, entropy, inverse variance, gray level nonuniformity, mean, IDN, kurtosis, skewness, and NESTD. Shape features did not show significant correlation to the treatment response.

### Prediction model building using machine-learning algorithms

Different weight planes for different DRFs were obtained from the SOM confirming that the candidate DRFs were not highly correlated. The SOM showed some degree of overlap in the candidate DRFs values between the two response groups. To determine whether the candidate DRFs, identified by the analysis of using the mixed-effect model and *t* test and confirmed by the self-organizing map (SOM), can be used to distinguish the good or bad response, they were used to build a response prediction model using a Bayesian regularization backpropagation neural network. Based on the network using the DRFs confirmed by the self-organizing maps, seven individual candidate DRFs (kurtosis, skewness, coarseness, NESTD, IDN, mean, and contrast) were identified to have the strongest correlation with the response, thus, could be used to build a response prediction model.

**Fig. 5:**
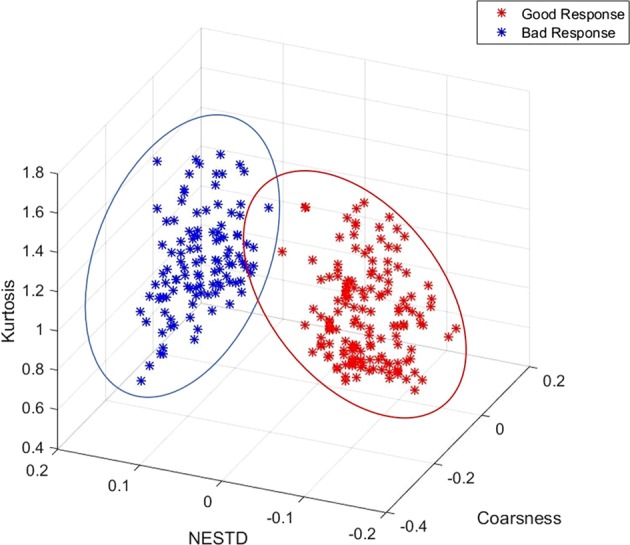
3D scatter plots for the best performing feature combination. The weekly DRFs for weeks 2–4 for 50 good responders (150 data points) and the 40 bad responders (120 data points) for the best performing features combinations (kurtosis–NESTD–coarseness) is displayed

The prediction model was improved by combining the DRFs. The best performing two-feature combination with cross validated area under the ROC curve (CV-AUC) of 0.92 was found to be for the kurtosis–coarseness combination. The performance increased to 94% using the kurtosis–coarseness–NESTD combination with an accuracy of 0.9. A similar performance (CV-AUC = 0.93) was obtained for the combination of Skewness–IDN–contrast. Table 1 summarizes the best performing two- and three-feature combinations, determined using the CV-AUC of the ROC curve, and their confidence intervals. Figure 5 shows 3D scatter plots of the best performing feature combinations (kurtosis–NESTD–Coarseness), including weekly DRFs of the 2–4 weeks for 50 good responders (150 data points) and 40 bad responders (120 data points). Using the external independent validation set (DRFs for weeks 2–4), the testing AUC was 0.96 for the Skewness–IDN–contrast and 0.98 for the kurtosis–coarseness–NESTD combination with an accuracy of 0.94. It is clear that these DRFs and their appropriate combinations can accurately predict the treatment response.

**Table 1:** The best performing two- and three-feature combinations as judged by the AUC and the confidence interval

Two-features combination	AUC	Confidence interval
Kurtosis, coarseness	0.92	[0.90, 0.95]
NESTD, coarseness	0.90	[0.89, 0.97]
Kurtosis, NESTD	0.89	[0.84, 0.91]
Kurtosis, skewness	0.88	[0.84, 0.92]
Two features combination	AUC	Confidence interval
Kurtosis, coarseness, NESTD	0.94	[0.91, 0.95]
Skewness, contrast, IDF	0.93	[0.91, 0.95]
Kurtosis, NESTD, skewness	0.82	[0.90, 0.95]
Kurtosis, skewness, mean	0.88	[0.85, 0.91]

## Discussion

As demonstrated in this work, delta radiomics is a quantitative method that can assess the treatment-induced net change of radiomic features over time and may be used for early prediction of treatment response during the treatment. A rigorous delta-radiomics analysis process to select appropriate DRFs to predict response was developed and demonstrated based on the 28 daily CT sets collected acquired during routine CT-guided CRT and pathological treatment response data for 90 pancreatic cancer patients. The process has identified appropriate delta-radiomic features that can predict treatment pathological response during the CRT delivery.

One limitation of delta radiomics is contour variations between the image sets that can affect the reproducibility of the results.^17^ Our newly developed NESTD map provided a useful tool to detect structure boundaries and to adjust the contours as needed for a more robust and reproducible feature extraction process. Another limitation, common for any radiomics work, is the restriction for using the data with certain technical and patient variations, e.g., from different scanners or different institutions, limiting the size of the data that can be used to train deep networks.^17^ For delta radiomics, however, DRFs are the relative values that may not strongly depend on certain imaging variations. For example, this study shows that variations in imaging parameters from two different scanners did not affect the DRF analysis. On the other hand, the data for patients with large amounts of motion or stent artifact that distort image quality needed to be excluded as they can introduce errors in DRFs. An exception is that certain features, which can have a low coefficient of variance, thus, can be less affected by the amount of motion, can still be identified as potential candidate DRFs.

The use of machine-learning algorithms appears to be an effective approach to build a response prediction model based on DRFs. Some potential problems can arise when implementing a neural network and limit its performance, such as local minima, overfitting, or classifying external independent data set as an outlier. In this study, the use of Bayesian regularization and Mahalanobis distance can account for these potential problems. The results reported here show promising initial results and demonstrate the need to further test this approach by using more patient data or data of other treatment or tumor sites. Larger data sets would improve the robustness of the neural network training using a variety of features including higher order features. More rigorous outcome data (e.g., local tumor control, survival) would help strengthen the correlation analysis. With further studies based on large and diversified patient data sets, the proposed delta-radiomics process may be developed as an invaluable tool to identify imaging biomarkers for individualized treatment, e.g., guide adaptive radiation therapy.

## Methods

### A general delta-radiomics process

**Fig. 6:**
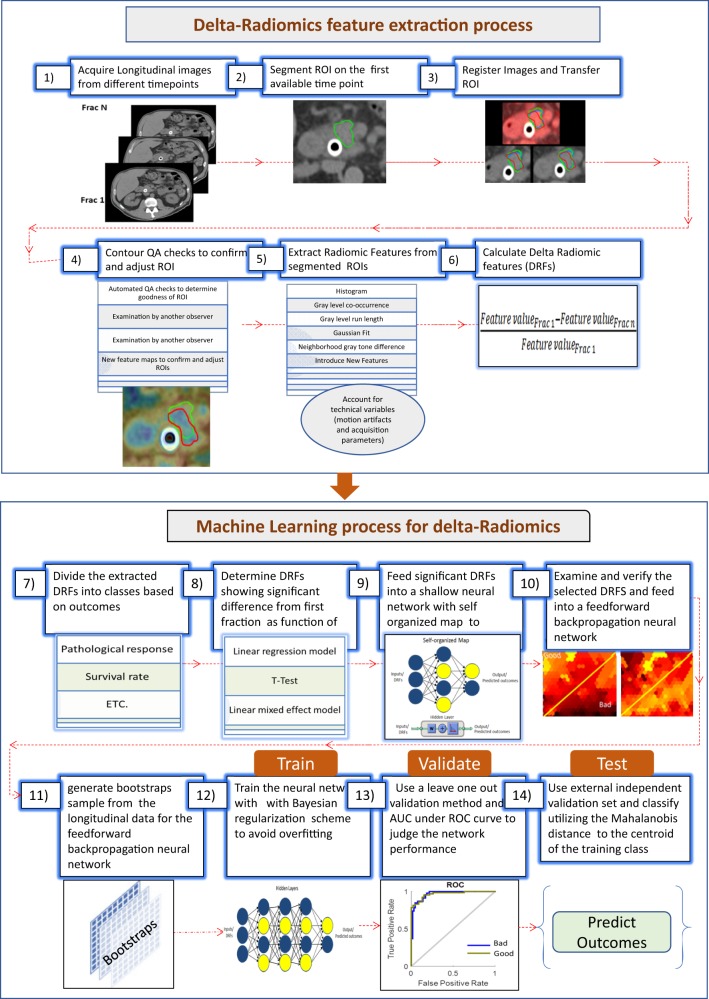
A general delta-radiomics process. Including extracting delta-radiomics features and building machine-learning models for treatment outcome prediction

We propose a delta-radiomic process based on machine learning. The process starts with acquiring a set of longitudinal images at different time points during treatment, followed with segmenting regions of interest (ROIs) on the image at the first time point. The longitudinal images are registered with each other either rigidly or deformably. The segmented ROIs are then populated to the images at the subsequent time points. The quality of the ROI contours is checked, either manually or automatically, to ensure their consistency over time. Radiomic features are extracted from the segmented ROIs from each time point and the changes in these radiomic features (i.e., DRFs) from those at the first time point are calculated. Due to the large number of radiomic features that can be extracted from the images, a Spearman correlation is used to rule out redundant DRFs. The selected DRFs are then tested to determine their significance as a function of treatment response using linear regression models, *t* test and mixed-effect models. Significant DRFs are further tested and modeled using machine-learning algorithms to create a model that can be used to predict outcome of a new patient. Figure 6 shows this general process for the delta-radiomics analysis. This process and the details of the machine-learning algorithms are further explained using the patient imaging and outcome data described below.

### Patient data

The proposed delta-radiomics process is demonstrated and tested by retrospectively analyzing the imaging and outcome data collected from 90 pancreatic cancer patients treated in our clinic in compliance with the relevant HIPPA guidelines and regulations. Written consent was waived for retrospective studies under the Medical College of Wisconsin IRB approval. All patients had resectable or borderline resectable pancreatic head tumor, and were treated with pre-operative chemotherapy with Folfirinox or Folfiri and concurrent CRT with gemzar or gemcitabine followed by surgery during the period between 2012 and 2017. All RT were delivered with daily guidance of non-contrast CTs acquired using two in-room CT scanners (installed in two RT rooms) immediately prior to each delivery. These daily CTs were analyzed. The patients had a median age of 67 years at the start of treatment, with 54% males and 46% females.

All patients underwent pancreatectomy after CRT with gross and microscopic pathology reported. The surgical specimen was fixed in formalin overnight, and then the pancreas was serially sectioned. The area of tumor and surrounding fibrosis in the pancreas were submitted for microscopic examination. Hematoxylin and eosin sections were prepared, and treatment effect was evaluated. A modified Ryan Scheme for tumor regression score recommended by the College of American Pathologists was used to evaluate treatment effect as follows: Grade 0 (G0): no viable cancer cells (complete response), Grade 1 (G1): single cells or small groups of residual cancer (near complete response), Grade 2 (G2): residual cancer with evident tumor regression, but more than single cells or rare small groups of cancer cells (partial response), and Grade 3 (G3): extensive residual cancer without evident regression (poor or no response).^23^ Among the 90 patients, there were 50 patients with good pathological response (G1 and G2), and 40 with bad pathological response (G3). These good- and bad-pathological response data were used to create the good and bad groups used in our analysis.

### CT acquisition parameters

To include more patient data while ensuring a robust model and exclude DRFs that are affected by acquisition parameter variations, the daily CT data were obtained from patients scanned using two Siemens CT scanners (in-room CTs) with slightly different acquisition parameters. The first group included daily CT data acquired from 50 patients using a CT scanner (Definition AS, Siemens), with a standard abdominal protocol consisting of the following parameters: 120 kVp tube voltage, 252 mA tube current, 0.5 s, 1.2 -mm focal spot, and standard filtered back-projection (FBP) algorithm with B30f kernels. The second group included daily CT data from 40 patients scanned using a different CT scanner (Emotion, Siemens) with another standard abdominal protocol employing the following parameters: 130 kVp tube voltage, 226 mA tube current, 0.6 s, 0.95 -mm focal spot, and standard FBP with B31s kernels. All analyzed CTs were reconstructed in a 512 × 512 × Z (slices) voxels with resolution 0.98 mm × 0.98 mm × 3 mm. To assess the effect of the acquisition parameters on radiomic feature selection, patients with the same pathological response were divided into two groups based on the two acquisitions protocols, and a multivariate regression analysis was used to determine the effect of acquisition protocols on the DRFs.

For each patient, daily CT sets were collected from the 28 treatment fractions during the delivery of CRT of 50.4 Gy in 28 daily fractions, resulting in a total of 2520 daily CT sets for analysis. For patients with respiratory motion >8 mm (*n* = 30), the daily CTs were acquired with respiratory gating, reducing the motion to below 3 mm (the residual motion in the gating window) during the CT acquisition. For each patient, daily CTs were registered rigidly with each other with manual adjustment, if necessary, to achieve the best local matching between the two CT sets. For each case, the contour of pancreatic head was delineated on the contrast-enhanced simulation CT and MRI and was populated to the CT of the first day (the first RT fraction), then to other daily CTs based on rigid image registration. The obtained contours were edited by experienced researchers using MIM software and verified independently by other experienced researchers to ensure consistency. In addition, these contours were checked using a new method proposed in this work (see below).

To minimize the effect of interfraction variation, daily CTs were registered locally based on the obtained contours. To estimate the effect of intrafraction (mostly respiratory) motions on DRFs, patients with the same pathological response were divided into two groups: low (<3 mm) and high (3–8 mm) motions, to examine the effect of motion on DRFs and to ensure that the DRFs selected for building the prediction model are not affected by the motions.

### Contour validation

Accurate and consistent segmentation between daily CTs is essential to improve the accuracy and reproducibility of the extracted DRFs. Since manual segmentation can vary significantly among even well-trained observers,^24^ it is desirable to develop a new method to reduce the segmentation variation. To do so, we introduced a new feature (normalized entropy to standard deviation difference, NESTD) that combines the GLCM “Entropy” feature and the histogram “Standard Deviation (STD)” feature. This new feature can be used to improve the detection of organ boundaries and to provide a way to standardize contour validation.

**Fig. 7:**
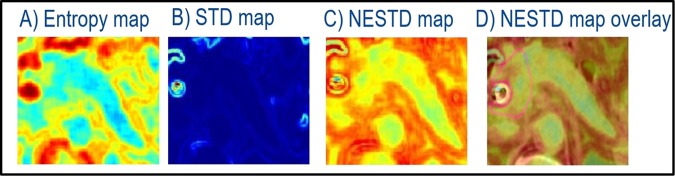
An example of NESTD map generation. **a** entropy map, **b** STD map, **c** the resultant NESTD map, and **d** the NESTD map overlaid on the original CT image

To obtain NESTD, a square ROI containing the pancreas and the surrounding tissue was defined for each CT slice. Normalized color encoded entropy and standard deviation maps were generated, using a script implementing MATLAB^®^ built-in functions (“entropyfilt” and “stdfilt”). For each map, the output pixel contains the feature value of a 3-by-3 neighborhood around the corresponding pixel in the input image. For pixels on the borders of the input image, a symmetric padding was used (i.e., the values of the padding pixels were a mirror reflection of the border pixels). The NESTD map was generated by taking the difference between the normalized entropy and standard deviation maps and was applied to detect the boundaries of different organs and to adjust the contours if necessary.^24^ Figure 7 shows an example of the entropy, standard deviation, and the resultant NESTD map for one CT slice. The same process was repeated for all the slices covering the pancreatic head. To evaluate the effectiveness of using the NESTD map for contour validation, the resultant map was overlaid on the original CT where the pancreas head was delineated. If the contour included tissue other than the pancreatic head, the contour would be adjusted. To obtain a feature value, the average of the values of the NESTD over the entire 3D tumor was used.

In this study, the contours generated manually were validated utilizing the NESTD map, and were adjusted if necessary. For example, the vessels that were not easily visualized on the CT and were included in the manual contour can be excluded by utilizing the new feature map.

### Delta-radiomics features

CT texture analysis was performed to extract over 1300 radiomic features from the segmented ROIs, using an available software package, IBEX^25^. Features extracted included intensity-based histogram, gray level run-length matrix (GLRNM)^26–32^, gray level co-occurrence matrix (GLCM)^27,28^, neighbor gray tone difference matrix (NGTDM)^29,33^, intensity histogram Gaussian fit, shape-based features,^27–30^ and our newly proposed NESTD feature.^24^

Since relatively high numbers of extracted radiomic features compared with the sample size can reduce the statistical power and increase the probability of data overfitting, a Spearman rank-order correlation coefficient was used to rule out low-rank redundant features (*r*_s_ > 0.9). To reduce directional dependence, GLCMs for a distance d = 1 and a particular direction were summed over the set of axial slices. These direction-specific matrices were then summed and averaged to create the final GLCM for the 3D ROI.^27,28^ Similarly, the GLRNMs were calculated in the 0 and 90 directions, and then summed and averaged to create a global 3D run-length matrix.^30–32,34,35^

The DRF of a radiomic feature at the *n*th fraction was calculated as the relative change of the feature value from its value at the first fraction, such that:

1 $${\mathrm{DRF}}n = \frac{{{\mathrm{Feature}}\;{\mathrm{value}}_{{\mathrm{Frac}}\;{\mathrm{1}}} - {\mathrm{Feature}}\;{\mathrm{value}}_{{\mathrm{Frac}}\;n}}}{{{\mathrm{Feature}}\;{\mathrm{value}}_{{\mathrm{Frac}}\;{\mathrm{1}}}}},{\mathrm{where}}\;n = {\mathrm{2:28}}$$

All DRFs were categorized based on the coefficient of variance (COV). The *p*-values of the *t* test and the modified signed-likelihood ratio test (MSLR) for equality of COV were calculated to assess the robustness of DRFs using R® software.

Spearman correlations, coefficient of variance (COV), and the modified signed-likelihood ratio test (MSLR) for equality of COV, and the *t* test, regression models, linear mixed-effects models that will be discussed in the next sections were built using R® built-in ggpubr, corrplot, lme4, datarim, cvequality packages, and functions.

### Machine-learning Algorithms

For the analysis of correlation between DRFs and pathological response, DRFs that were not prone to motion variation and/or acquisition parameters (due to the choice of acquisition parameters) were used in a machine-learning process. This process started with dividing the data set into two groups, good- (50 cases with 28 fractions each) and bad- (40 cases with 28 fractions each) response groups, based on their pathology response to CRT. To determine potential DFRs that could correlate to treatment response, DRFs were evaluated to determine when they started to change during the treatment as compared with their values at the first fraction and if these changes were different between the patients with good and bad responses. A metric trend was established using a linear regression model to find the best fit for each feature versus response and determine those with potential trends. A *t* test was performed to determine which DRFs changed significantly between the two response groups for the entire course of treatment, between two time points (e.g., daily, weekly) to pinpoint the time at which significantly different changes between the two response groups occurred. Features were also evaluated to determine if they changed during the treatment by fitting linear mixed-effects models using R^®^ software for the DRFs as a function of response with two random effects as follows:

2 $$\left\lceil {{\mathrm{model}} = lme\left( {\mathrm{{DRF}}} \right.\sim {\mathrm{Response}} + \left( {{\mathrm{1}}\left| {{\mathrm{Patient}}} \right.} \right) + \left( {{\mathrm{1}}\left| {{\mathrm{Fraction}}} \right.} \right)} \right\rceil$$

The first random effect assumes a different intercept for each patient to account for patient-dependent variation. The second random effect accounts for the fractional-dependent variation from the longitudinal study. The *p*-value of the log likelihood ratio for each model was calculated. The features that showed a trend and passed both the linear mixed-effect model and the *t* test (*p* < 0.05) were selected to be used to build a model to predict treatment response as early as possible during treatment.

To confirm that the DRFs selected by the *t* test, regression model and the linear mixed-effect model are appropriate and to ensure these features are not highly correlated to each other, a self-organizing neural network^36^ was built using the Matlab built-in neural clustering app to cluster the data based on the similarity while considering clustering in multiple dimensions. The training was performed using a batch algorithm, the slope of the DRFs over time for each data set was presented to the network before any weight was updated. The algorithm then determined a winning neuron for each input vector. Each of the weight vectors was updated such that it moves to the average position of all input vectors for which it or a neighbor neuron was a winner. Using self-organizing map, the weight plane of each feature used in the training process can be visualized. This can provide us with information regarding the potential of using a feature to discriminate between the two response groups. If the weight plan of two inputs is similar, then these inputs are highly correlated. This was used to assure that the features included are not highly correlated and to confirm previous analysis.

To identify DRFs with the highest prediction power, a feedforward backpropagation neural network was utilized. The good- and bad-response groups were divided into training and testing sets. For each feature, the daily DRFs for 50 patients (30 good- and 20 bad-responders with 28 fraction each, i.e., 840 (30 × 28) data points for good- and 560 (20 × 28) data points for bad-response group) were computed and used for the training. Combinations of either two or three of the DRFs that were previously selected using the *t* test and linear mixed-effect model, and were confirmed to be less correlated using the self-organizing map were used. Multiple models were examined using all possible two and three DRF combinations at a time. For each model, the neural network inputs (two or three daily DRFs) were nonlinearly mapped to three hidden neurons which were linearly mapped to an output variable.^37^

However, two potential problems arise when optimizing a neural network, (1) a local minimum, where optimization terminate at a local not global minimum of the cost function, and (2) overfitting, where the model fits the noise in the training set. Generally, regularization by adding weight penalty terms to the cost function can help avoid these potential problems. However, it can be computationally intensive if the weights penalty is determined by validation. To efficiently determine the weight penalty parameters, a Bayesian regularization scheme (using “trainbr” function) was added to the neural network in the training process, leading to Bayesian neural network (BNN) models.^37–40^

By introducing Bayesian inference in the neural network, the optimal weight penalty parameter is estimated by a Bayesian approach to compute the likelihood of class membership given some characteristic of that class such that,^41–44^

3 $$p\left( {w_{\mathrm{j}}{\mathrm{|}}t} \right) = \frac{{p\left( {t{\mathrm{|}}w_{\mathrm{j}}} \right)p(w_{\mathrm{j}})}}{{p(t)}}$$

where, *p* (*t*|*w*_j_) is the class conditioned probability or likelihood of target *t* given model parameter *w*, *p* (*w*_j_) is a prior probability, *p*(*t*) is an evidence (usually ignored), and *p*(*w*_j_|*t*) is the measurement conditioned or the posterior probability

In a Multivariate Normal Bayesian Classification, given multiple classes, each class *w*_j_ has its own mean vector *m*_j_ and covariance matrix *c*_j_, such that the class-conditional probabilities are

4 $${{p}}\left( {{{t|w}}_{{{\mathrm{j}}}}} \right) = 2{{\pi }}^{ - {{d}}/2}\left| {{{c}}_{{{\mathrm{j}}}}} \right|^{ - 1/2}{{{\mathrm{exp}}}} - 1/2\;({{t}} - {{m}}_{{{\mathrm{j}}}})^{\mathrm{T}}{{c}}_{\mathrm{j}}^{ - 1}({{t}} - {{m}}_{\mathrm{j}})$$

To move from probabilities to discriminants we need to maximize: *p*(*w*_j_|*t*) or *p*(*t*|*w*_j_) *p*(*ω*_j_) or log(*p*(*t*|*ω*_j_)) + log((*P*(*ω*_j_)) and to link classical neural network with Bayesian statistics, minus the log likelihood is defined.^42^ In other words, we need to maximize

5 $$- \left({{\log}}\, p( {t|w_{\mathrm{j}}} ) - 1/2\;{{\log}}\left| {c_{\mathrm{j}}} \right| - 1/2(t - m_{\mathrm{j}})^{T}c_{\mathrm{j}}^{- 1}( {t-m_{\mathrm{j}}} )\right)$$

However, even with Bayesian regularization nonlinear neural network model, performance can be reduced when the model is evaluated over independent testing set. One possibility is that some values among the test data can be considered outliers relative to the training data used in building the models. To overcome this potential problem, Mahalanobis distance can be used to consider the mean and the covariance matrix of each class, and hence, its spread in the multidimensional space.^38^

In other word from Eq. 5, the expression $- {\mathrm{1/2}}/(t - {m}_{\mathrm{j}})^Tc_{\mathrm{j}}^{ - {\mathrm{1}}}(t - m_{\mathrm{j}})$ can be thought of as $\left\| {\left( {t - m_{\mathrm{j}}} \right.} \right\|^2c_{\mathrm{j}}^{ - {\mathrm{1}}}$ which looks like a squared distance multiplied by the inverse covariance matrix (*c*), that acts as a metric (stretching factor) on the space. Thus, using the Mahalanobis distance is equivalent to maximizing the likelihood *p*(*t*|*w*_j_) used in Bayesian statistics.^38^ Classification using BNN models incorporating the minimum (Mahalanobis) distance classifier (using “Mahal” function) can reduce the number of the data points classified as outliers. The results of the classification were the class with the highest probability (i.e., minimum distance to the centroid of the trained class as defined by the mean and covariance matrix of the model).

In our analysis for the training set, cross-validation (using “cvpartition” function) was performed using a leave-one-out method (one patient, with its all daily DRFs, is left out). Testing was performed using external independent validation sets of the daily DRFs from 40 patients not used for the training (20 good- and 20 bad-responders with 28 fraction each). A total of 120 weekly DRFs values from weeks 2–4 during the treatment were used to examine the performance of BNN. Classification was done using the minimum Mahalanobis distance to the centroid of the training class. The performance of each model was judged using the AUC. For each DRFs’ combination examined, to obtain the confidence intervals for the AUC, bootstraps sampling (using “bootstrp” function) was utilized with nboot = 100, i.e., from each bootstrap sample data one BNN model was trained, yielding 100 BNN models, and the ensemble mean of the resulting 100 BNN models was used as the final BNN model.

## Supplementary information

Supplemental Materials

## Data Availability

Data relating to the weekly DRFs for weeks 2–4 for the 50 good responders (150 data points) and the 40 bad responders (120 data points) for the best-performing feature combinations (kurtosis, NESTD, and coarseness) that are used to generate the 3D plots, table showing the mean and standard deviation per fraction from all patients in the same response group for the relative net change of volume and sphericity for the good and bad response groups, figures showing the average changes over time for volume and sphericity showing overlap between the two response groups until the last week of treatment, which suggest that these features are not very useful for early prediction of treatment response, box plots of features showing significant differences between the two response groups (mean, IDN, entropy, and NESTD) combining all fractions and all patients in each response group, box plots of weekly change of a feature showing significant difference (kurtosis) and a feature not showing significant difference (IQR) for all patients and all fractions in the good and bad response groups, confusion matrix for the training and the external independent validation sets using the combination of kurtosis, coarseness, and NESTD features are available in the supplementary materials. Data are available from the corresponding author upon reasonable request.

## Code availability

Sample Matlab and R scripts are available in the supplementary materials. For our analysis, the pancreatic head was segmented in MIM 6.7.6 software (available at https://www.mimsoftware.com/). Radiomic features were extracted using IBEX software standalone version (available at https://www.dropbox.com/sh/tjk28ti5btmtos4/AAD9pkpfgmzlDYU-3TMCR0a2a). MATLAB scripts were written with built-in MATLAB function in MATLAB R2015a version (available at https://www.mathworks.com/products/matlab.html). Codes are available from the corresponding author upon reasonable request.
