# Supplementary material for: A machine learning based delta-radiomics process for early prediction of treatment response of pancreatic cancer
Source: NPJ Precis Oncol. 2019 Oct 4;3:25. doi: 10.1038/s41698-019-0096-z (PMC6778189; doi:10.1038/s41698-019-0096-z)
Supplement: Supplementary file 1 — Supplemental Materials [file 41698_2019_96_MOESM1_ESM.pdf]

Supplementary Methods

For our analysis, the Pancreatic head was segmented in MIM 6.7.6 software available at (https://www.mimsoftware.com/). Radiomic features were extracted using IBEX software standalone version available at (https://www.dropbox.com/sh/tjk28ti5btmtos4/AAD9pkpfgmzlDYU-3TMCR0a2a).

Features extracted included intensity-based histogram, gray level co-occurrence matrix, neighbor gray tone difference matrix, gray level run length matrix, Intensity histogram Gaussian fit, and Shape-based features. Histogram features were calculated from the full distribution of intensities in the ROI. Examples of features obtained from this matrix included mean, standard deviation, skewness, kurtosis, median, minimum, maximum, etc. The GLCM features are second order statistics that quantify the frequency at which gray-level intensity appears adjacent to other gray-level intensity in a particular direction, thus representing the spatial information of the image. In other words, the GLCM represents the second order joint conditional probability density function of an image. It is calculated in different 2D directions (e.g., 0, 45, 90, and 135) for each axial slice and covers connected directions of neighboring voxels in 3D space. To include data from all connected directions, avoid directional dependence, and reduce the number of features that can be used for our analysis, GLCMs for a distance d=1 and a particular direction are summed over the set of axial slices. These direction-specific matrices are then summed and averaged to create the final GLCM for the 3D ROI. Examples of features obtained from this matrix included entropy, inverse difference normalized, inverse variance, inverse difference moment normalized, cluster shade, cluster prominence, cluster tendency, autocorrelation, difference entropy, dissimilarity, correlation, energy, global uniformity, homogeneity, information measure, maximum probability, etc. The GLRLM is calculated for a particular direction and defines the consecutive image pixels that have the same gray level intensity value. In this study, the GLRMs were calculated in the 0 and 90 directions and then summed and averaged to create a global 3D run-length matrix. Examples of features obtained from this matrix included short run emphasis, run percentage, short run high gray level emphasis, run length nonuniformity, low gray level emphasis, etc. The NGTDM quantifies the human perception of five visual characteristics: coarseness, contrast, busyness, complexity, and texture strength. The average difference between each pixel and its neighboring pixels (5x5) is calculated. Thus, the peripheries of the ROI do not contribute directly to it. These average differences are then summed for each pixel of the same gray-level intensity and summarized in a one-column matrix with an entry for each gray-level in the segmented region. Intensity histogram Gaussian fit features describe the mean, area, and standard deviation of a Gaussian fitted to the extracted histogram using MATLAB default fit function. Shape-based features describe the geometric shape of the tumor, maximum 3D diameter, sphericity, orientation, compactness, spherical disproportion, volume and surface to volume ratio. For instance, the maximum 3D diameter is measured as the largest pairwise Euclidian distance between voxels on the surface of the tumor volume; the surface area is calculated by triangulation; and the volume is

determining by counting the number of pixels in the tumor region and multiplying this number by the voxel size.

Our newly developed NESTD feature (defined as the normalized entropy to standard deviation difference on a voxel by voxel basis) was calculated using the following MATLAB scripts written with built-in MATLAB function in MATLAB R2015a version available from (https://www.mathworks.com/products/matlab.html).

```
%%%%%%%%%%%%%%%%%%%%%%%%%%%%%%%%%%%%%%%%%%%%%%%%%%%%%%%%%
%NESTD MAP
%%%%%%%%%%%%%%%%%%%%%%%%%%%%%%%%%%%%%%%%%%%%%%%%%%%%%%%%%
imgheaders = cell(1, length(CT_images));
for i  = 1:length(CT_images)
     info = dicominfo(strcat(imagedir, CT_images(i).name));
     i=i+1
end

imgheaders = info(1:length(filescount))
Mat_X=size(dicomread(strcat(imagedir,CT_images(1).name)),2);
Mat_Y=size(dicomread(strcat(imagedir,CT_images(1).name)),1);
info1=dicominfo(strcat(imagedir,CT_images(1).name));
info2=dicominfo(strcat(imagedir,CT_images(filescount).name));

 images_init(:,:,1)=dicomread(strcat(imagedir,CT_images(1).name));
      try
        images_HU(:,:,1)=images_init(:,:,1)*info1.RescaleSlope +
info1.RescaleIntercept;
        end
    images(:,:,1)=images_init(:,:,1);
    infoI=dicominfo(strcat(imagedir,CT_images(1).name));
    z(1) = infoI.ImagePositionPatient(3);
        images_scaled(:,:,1) = mat2gray(images_init(:,:,1));
  I=images_scaled(:,:,1);
I=double(I);
J= entropyfilt(I);
K= stdfilt(I);
JN=(J-(min(min(J)))./(max(max(J))-min(min(J)));
KN=(K-(min(min(K)))./(max(max(K))-min(min(K)));
D=JN-KN;
figure
imshow(D,[])
colormap('jet')
colorbar
title('NESTD ')
```

Spearman correlations, coefficient of variance (COV), and the modified signed-likelihood ratio test (MSLR) for equality of COV t-test, regression models, linear mixed effects models were built using R® software available from (https://cran.case.edu/). R software built-in ggpubr, corrplot, lme4, datarim, cvequality packages were used.

```
#########################
install.packages("corrplot")
library(corrplot)
install.packages("PerformanceAnalytics")
library("PerformanceAnalytics")
install.packages("ggpubr")
install.packages("datarium")
install.packages("cvequality")
install.packages("lme4")
library(knitr)
library(ggpubr)
library(ggplot2)
library(lme4)
library(ggbeeswarm)
library(cvequality)

my_data <- read.csv(file.choose())
p <- ggplot(my_data, aes(x=status, y=Kurt, color=status)) +
  geom_boxplot()
p + stat_summary(fun.y=mean, geom="point", shape=23, size=4)
p <- ggboxplot(my_data, x = "Group", y = "ClusterTend", color = "Group",
palette = "npg",facet.by = "Week", short.panel.labs = FALSE)
p + stat_compare_means(method = "t.test")
######
res <- cor(my_data)
round(res, 2)
cor(res, use = "complete.obs")
res2 <- rcorr(as.matrix(my_data))
res2
res2$r
res2$P
res2<-rcorr(as.matrix(my_data))
flattenCorrMatrix(res2$r, res2$P)
symnum(res2, abbr.colnames = FALSE)
col <- colorRampPalette(c("blue", "white", "red"))(20)
heatmap(x = res, col = col, symm = TRUE)
col <- colorRampPalette(c("#BB4444", "#EE9988", "#FFFFFF", "#77AADD",
"#4477AA"))
corrplot(res, method="color", col=col(200),
         type="upper", order="hclust",
         addCoef.col = "black", # Add coefficient of correlation
         tl.col="black", tl.srt=45, #Text label color and rotation
         # Combine with significance
         p.mat = p.mat, sig.level = 0.05, insig = "blank",
         # hide correlation coefficient on the principal diagonal
         diag=FALSE
         )
#####
ggplot(my_data, aes(x = Kurtosis, y = Response)) + geom_point() +
stat_smooth()
cor(my_data $ Response, my_data $ Kurtosis)
```

```
model <- lm (Kurtosis ~ Response, data = my_data)
model
summary(model)
confint(model)

######
my_data= read.csv(file.choose( ))
Kurtosis.null = lmer(dKurtosis ~ Response+ (1|Response),
data=my_data,REML=FALSE)

Kurtosis.model = lmer(dKurtosis ~ Response +(1+fraction|subject),
data=my_data,REML=FALSE)
anova(Kurtosis.null,Kurtosis.model)
anova(Kurtosis.model)
coef(Kurtosis.model)
summary(Kurtosis.model)
boxplot(dKurtosis~ Week*Response, col=c("white","lightgray"),my_data)
p<-ggboxplot(my_data, x = "Response", y = "dKurtosis",palette =
"jco",facet.by ="fraction", short.panel.labs = FALSE)
p + stat_compare_means(label = "p.format",
method="t.test")+stat_summary(fun.y=mean, geom="point", shape=23, size=3)

p<-ggboxplot(my_data, x = "Response", y = "dKurtosis",palette =
"jco",facet.by ="Week", short.panel.labs = FALSE)
p + stat_compare_means(label = "p.format",
method="t.test")+stat_summary(fun.y=mean, geom="point", shape=23, size=3)
########
Data <- read.table(".\\Kurtosis Motion.txt", sep=",", header=TRUE);
p <- ggboxplot(Data, x = "Motion", y = "Kurtosis",color = "Motion", palette =
"jco")
p + stat_compare_means(Motion = "p.format")
p <- ggplot(Data, aes(x=Motion, y=Kurtosis, fill=Motion)) +
geom_boxplot()+labs(title="Motion effect on Kurtosis",x="Motion", y =
"Kurtosis")
p+stat_compare_means(label="p.format", method = "t.test")
p+stat_compare_means(label = "p.format", method =
"t.test")+stat_summary(fun.y=mean, geom="point", shape=23, size=4)

Kurtosis_Variation_by_Motion_cv_test_MSLRT <- data.frame(`Test name` =
c("asymptotic",  "M-SLRT"), `Test statistic` =
c(Kurtosis_Variation_by_Motion_cv_test$D_AD,Kurtosis_Variation_by_Motion_cv_t
est_MSLRT$MLRT), `p-value` =
c(Kurtosis_Variation_by_Motion_cv_test$p_value,Kurtosis_Variation_by_Motion_c
v_test_MSLRT$p_value),    check.names = FALSE)
```

3D plots were created in MATLAB 2015a version. Self-organized map was created using MATLAB built-in neural clustering app. The neural network with Bayesian regularization scheme

was built using MATLAB built-in “trainbr” function. MATLAB scripts are shown in the subsequent section.

```
%%%%%%%%%%%%%%%%%%%%%%%%%%%%%%
figure
plot3(dmeasdelta(1:150,3), dmeasdelta(1:150,2), dmeasdelta(1:150,1),'r*')
hold on
plot3(dmeasdelta(151:end,3), dmeasdelta(151:end,2),
dmeasdelta(151:end,1),'b*')
grid on
xlabel('Coarsness')
ylabel('NESTD')
zlabel('Kurtosis')
legend('Good Response', 'Bad Response')

%%%%%%%%%%%%%%%%%%%%%%%%%%%%%%
x=x';
t=t';
var_t=mean(var(t',1)); %t variance
[inputs,obs]=size(x); %
hiddenLayerSize =3; %max number of neurons
NN = cvpartition(t','leaveout',1);; % number of training runs
neurons = [1:hiddenLayerSize]';
numNN=NN.NumTestSets;
training_no = 1:nn;
obs_no = 1:obs;
nets = cell(hiddenLayerSize,numNN);
trainOutputs = cell(hiddenLayerSize,numNN);
valOutputs = cell(hiddenLayerSize,numNN);
testOutputs = cell(hiddenLayerSize,numNN);
Y_all = cell(hiddenLayerSize,numNN);
performance = zeros(hiddenLayerSize,numNN);
trainPerformance = zeros(hiddenLayerSize,numNN);
valPerformance = zeros(hiddenLayerSize,numNN);
testPerformance = zeros(hiddenLayerSize,numNN);
e = zeros(numNN,obs);
e_all = cell(hiddenLayerSize,numNN);
NMSE = zeros(hiddenLayerSize,numNN);
r_train = zeros(hiddenLayerSize,numNN);
r_val = zeros(hiddenLayerSize,numNN);
r_test = zeros(hiddenLayerSize,numNN);
r = zeros(hiddenLayerSize,numNN);
Rsq = zeros(hiddenLayerSize,numNN);
[bootstat,x] = bootstrp(1000,@corr,x,t);
for j=1:hiddenLayerSize
    for i=1:training_no
        trIdx = NN.training(i);
        teIdx = NN.test(i);
        xtrain=x(trIdx,:);
          ytrain=t(trIdx,:);
          xtest=x(teIdx,:);
          ytest_true=t(teIdx,:);
      trainFcn = 'trainbr';  % Bayesian Regularization backpropagation.
      % Create a Fitting Network
      net = fitnet(j,trainFcn);
      net.input.processFcns = {'removeconstantrows','mapminmax'};
```

```
      net.output.processFcns = {'removeconstantrows','mapminmax'};
      net.divideFcn = 'divideind';
   net.divideMode = 'sample';
   mse_goal = 0.01*var_t;
      net.performFcn = 'mse';
      net.trainParam.goal = mse_goal;
      net.plotFcns = {'plotperform','plottrainstate','ploterrhist', ...
          'plotregression', 'plotfit', 'plotroc'};
      for i=1:numNN
        % Train the Network
        net = configure(net,xtrain,ytrain);
        disp(['No. of hidden nodes '  num2str(j)  ', Training ' num2str(i)
'/' num2str(numNN)])
        [nets{j,i}, tr{j,i}] = train(net,xtrain,ytrain);
        y = nets{j,i}(xtrain);
        e = gsubtract(ytrain,y);
        e_all{j,i}= e;
        trainTargets = t .* tr{j,i}.trainMask{1};
        valTargets = t .* tr{j,i}.valMask{1};
        testTargets = t .* tr{j,i}.testMask{1};
        trainPerformance(j,i) = perform(net,trainTargets,y);
        valPerformance(j,i) = perform(net,valTargets,y);
        testPerformance(j,i) = perform(net,testTargets,y);
        performance(j,i)= perform(net,xtrain,ytrain);
        rmse_train(j,i)=sqrt(trainPerformance(j,i));
        rmse_test(j,i)=sqrt(testPerformance(j,i));
        rmse(j,i)=sqrt(performance(j,i));
        Y_all{j,i}= y;
        trainOutputs {j,i} = y .* tr{j,i}.trainMask{1};
       valOutputs {j,i} = y .* tr{j,i}.valMask{1};
        testOutputs {j,i} = y .* tr{j,i}.testMask{1};
        [r] = regression(t,y);
        [r_train] = regression(trainTargets,trainOutputs{j,i});
        [r_val(j,i)] = regression(valTargets,valOutputs{j,i});
        [r_test] = regression(testTargets,testOutputs{j,i});
        NMSE = mse(e_all{j,i})/mean(var(t',1));
        Rsq = 1-NMSE;
      end
      [minperf_train,I_train] = min(trainPerformance',[],1);
      minperf_train = minperf_train';
      I_train = I_train';
   [minperf_val,I_valid] = min(valPerformance',[],1);
     minperf_val = minperf_val';
     I_valid = I_valid';
     a=find(strcmp(ytrain,'Good' ));
          count=length(a);
          xtrainf=xtrain(1:count,:);
          xtrainc=xtrain((count+1):end,:);
          df=mahal(xtest,xtrainf);
          dc=mahal(xtest, xtrainc);
          if df> dc
              d=dc;
              ytest2='Bad';
          else
               d=df;
               ytest2='Good';
          end
```

```
          if strcmp(ytest_true,'Good')
              ytest_trueAll(i)=1;
          else
              ytest_trueAll(i)=0;
          end
          if strcmp(ytest2,'Bad')
              ytest_All(i)=1;
          else
              ytest_All(i)=0;
          end

      end
      %Compute True positive rate
          logic_bad= (ytest_trueAll==1);
          ytest_Expbad=ytest_All(logic_bad);
          TPR = sum(ytest_Expbad==ytest_trueAll(logic_bad))/sum(logic_bad);
       %Compute True negative rate
          logic_good= (ytest_trueAll==0);
          ytest_Expgood=ytest_All(logic_good);
          TNR =
sum(ytest_Expgood==ytest_trueAll(logic_good))/sum(logic_good);
           %Compute false positive rate
          FPR=1-TNR;
           ROC=[ROC; [FPR TPR]];
TPR_all=ROC(:,2);
FPR_all=ROC(:,1),
AUC= sum( diff(TPR_all).*diff(FPR_all)/2) + sum( TPR_all(1:end-
1).*diff(FPR_all)) + FPR_all(1)*TPR_all(1)/2;
      [minperf,I_perf] = min(performance',[],1);
      minperf = minperf';
      I_perf = I_perf';
      [maxRsq,I_Rsq] = max(Rsq',[],1);
      maxRsq = maxRsq';
      I_Rsq = I_Rsq';
      [train_min,train_min_I] = min(minperf_train,[],1);
    [val_min,val_min_I] = min(minperf_val,[],1);
      [test_min,test_min_I] = min(minperf_test,[],1);
      [perf_min,perf_min_I] = min(minperf,[],1);
      [Rsq_max,Rsq_max_I] = max(maxRsq,[],1);
end
```

Supplementary Table

Table 1: Data of the weekly DRFs for weeks 2-4 for 50 good responders (150 data points) and the 40 bad responders (120 data points) for the best performing features combinations (kurtosis-NESTD-Coarseness).

Kurtosis	NESTD	Coarseness	Response	Kurtosis	NESTD	Coarseness	Response
0.8	-0.09	0.03	Good	0.54	0.18	0.003	Bad
0.83	-0.1	0.029	Good	0.58	0.18	0.003	Bad
1.29	-0.04	0.027	Good	0.75	0.18	0.028	Bad
0.87	-0.07	0.03	Good	0.53	0.2	0.156	Bad
0.72	-0.15	0.032	Good	0.45	0.2	0.2	Bad
1.01	-0.1	0.029	Good	0.84	0.17	-0.052	Bad
1.03	-0.09	0.029	Good	1.07	0.19	0.086	Bad
0.73	-0.09	0.029	Good	0.64	0.18	-0.019	Bad
0.82	-0.09	0.03	Good	0.71	0.18	0.014	Bad
0.83	-0.03	0.028	Good	0.7	0.19	0.06	Bad
0.86	-0.13	0.029	Good	0.71	0.18	-0.013	Bad
0.9	-0.13	0.03	Good	0.79	0.17	-0.072	Bad
0.76	-0.12	0.029	Good	0.79	0.17	-0.078	Bad
0.77	-0.16	0.032	Good	1.12	0.14	0.065	Bad
0.59	-0.04	0.028	Good	1.03	0.12	-0.142	Bad
0.43	-0.04	0.026	Good	0.86	0.13	-0.062	Bad
0.49	-0.06	0.027	Good	0.87	0.14	-0.021	Bad
0.42	-0.11	0.028	Good	1.23	0.15	0.094	Bad
0.47	-0.1	0.027	Good	1.05	0.13	-0.054	Bad
0.49	-0.08	0.026	Good	1.15	0.14	0.016	Bad
0.46	-0.08	0.026	Good	0.78	0.15	0.101	Bad
0.49	-0.09	0.026	Good	0.64	0.13	-0.056	Bad
0.53	-0.08	0.027	Good	0.82	0.13	-0.062	Bad
0.64	-0.09	0.026	Good	0.94	0.14	0.059	Bad
0.48	-0.12	0.027	Good	0.97	0.13	-0.036	Bad
0.56	-0.1	0.028	Good	0.87	0.15	0.125	Bad
0.51	-0.09	0.026	Good	0.96	0.12	-0.13	Bad
0.54	-0.09	0.028	Good	0.79	0.14	0.051	Bad
0.49	-0.07	0.029	Good	0.81	0.15	0.182	Bad
0.51	-0.11	0.028	Good	0.99	0.13	-0.063	Bad
0.56	-0.07	0.028	Good	0.6	0.13	-0.067	Bad
0.65	-0.07	0.027	Good	0.85	0.18	-0.029	Bad
0.5	-0.09	0.027	Good	0.83	0.18	-0.059	Bad

0.63	-0.06	0.014	Good	0.85	0.11	0.066	Bad
0.54	-0.04	0.015	Good	1.2	0.17	0.027	Bad
0.64	-0.11	0.016	Good	0.69	0.18	-0.097	Bad
0.55	-0.06	0.015	Good	1.01	0.09	-0.061	Bad
0.42	-0.03	0.015	Good	1.08	0.08	-0.134	Bad
0.44	-0.03	0.015	Good	0.75	0.1	0.026	Bad
0.53	-0.01	0.015	Good	0.88	0.1	0.036	Bad
0.47	-0.02	0.015	Good	1.35	0.1	0.039	Bad
0.53	-0.01	0.015	Good	0.72	0.19	-0.087	Bad
0.59	-0.04	0.016	Good	0.81	0.1	0.024	Bad
0.48	-0.02	0.014	Good	0.8	0.18	-0.084	Bad
0.55	-0.08	0.015	Good	1.5	0.17	-0.087	Bad
0.51	-0.05	0.016	Good	0.97	0.1	0.047	Bad
0.56	-0.07	0.015	Good	1.4	0.11	-0.019	Bad
0.5	-0.03	0.014	Good	0.87	0.11	-0.037	Bad
0.62	-0.06	0.016	Good	1.03	0.1	-0.025	Bad
0.62	-0.03	0.016	Good	0.86	0.11	-0.002	Bad
0.55	-0.05	0.009	Good	0.83	0.1	0.002	Bad
0.72	-0.05	0.009	Good	0.8	0.1	0.067	Bad
1.12	-0.06	0.009	Good	1.1	0.11	0.055	Bad
0.59	-0.04	0.009	Good	0.9	0.11	0.05	Bad
0.54	-0.04	0.009	Good	0.89	0.11	-0.061	Bad
0.56	-0.03	0.009	Good	1.12	0.12	-0.161	Bad
0.51	-0.04	0.01	Good	0.76	0.11	-0.064	Bad
0.53	-0.01	0.009	Good	0.92	0.1	0.075	Bad
0.59	-0.04	0.01	Good	0.82	0.19	0.132	Bad
0.68	-0.02	0.01	Good	0.85	0.18	-0.011	Bad
0.52	-0.04	0.01	Good	1.04	0.1	0.14	Bad
0.53	-0.04	0.01	Good	1.11	0.12	0.052	Bad
0.51	-0.05	0.01	Good	0.75	0.12	0.117	Bad
0.5	-0.06	0.01	Good	0.96	0.11	0	Bad
0.58	-0.03	0.009	Good	1.08	0.12	-0.004	Bad
0.54	-0.05	0.01	Good	0.7	0.11	0.013	Bad
0.59	-0.05	0.011	Good	1.1	0.12	0.093	Bad
0.62	-0.04	0.01	Good	0.81	0.11	0.013	Bad
0.94	-0.04	0.009	Good	0.86	0.11	-0.08	Bad
0.84	-0.03	0.015	Good	0.78	0.12	-0.014	Bad
0.8	-0.05	0.015	Good	1.08	0.16	0.047	Bad

1.26	0.02	0.016	Good	0.69	0.15	-0.006	Bad
0.71	-0.01	0.016	Good	1.04	0.15	0.015	Bad
0.97	-0.02	0.017	Good	1.31	0.16	-0.12	Bad
0.93	-0.01	0.016	Good	1.32	0.16	0.057	Bad
0.8	-0.04	0.015	Good	1.08	0.14	-0.034	Bad
0.81	-0.03	0.016	Good	1.03	0.16	0.013	Bad
1	0.01	0.016	Good	0.74	0.14	0.126	Bad
0.88	-0.03	0.015	Good	0.95	0.15	0.079	Bad
0.91	0	0.016	Good	0.96	0.16	-0.02	Bad
0.9	-0.03	0.017	Good	0.97	0.17	0.011	Bad
0.67	-0.01	0.016	Good	0.93	0.16	0.019	Bad
0.85	-0.03	0.016	Good	0.82	0.17	0.026	Bad
0.87	-0.03	0.017	Good	0.91	0.13	0.113	Bad
0.71	-0.06	0.015	Good	0.85	0.14	0.054	Bad
0.72	-0.04	0.015	Good	0.69	0.14	-0.053	Bad
0.82	-0.02	0.016	Good	0.84	0.11	-0.029	Bad
0.6	-0.11	0.011	Good	0.45	0.2	0.2	Bad
0.75	-0.09	0.011	Good	0.47	0.18	-0.029	Bad
0.6	-0.04	0.01	Good	0.45	0.18	-0.037	Bad
0.68	-0.06	0.01	Good	0.5	0.17	-0.119	Bad
0.94	-0.02	0.018	Good	0.55	0.18	-0.03	Bad
0.91	-0.03	0.018	Good	1.1	0.18	-0.014	Bad
0.91	-0.06	0.018	Good	0.53	0.2	0.156	Bad
0.88	-0.07	0.017	Good	0.53	0.18	0.018	Bad
1	-0.02	0.018	Good	1.06	0.18	0.02	Bad
0.98	-0.06	0.018	Good	0.49	0.17	-0.06	Bad
1.01	-0.08	0.018	Good	0.91	0.18	0.01	Bad
0.89	-0.05	0.018	Good	0.57	0.15	-0.223	Bad
0.97	-0.07	0.018	Good	0.78	0.17	-0.062	Bad
0.91	-0.11	0.017	Good	0.84	0.17	-0.052	Bad
0.91	-0.08	0.018	Good	0.84	0.17	-0.052	Bad
0.88	-0.1	0.018	Good	0.79	0.17	-0.072	Bad
0.79	-0.06	0.018	Good	0.79	0.17	-0.078	Bad
0.83	-0.11	0.019	Good	1.03	0.12	-0.142	Bad
1.06	-0.08	0.018	Good	1.47	0.13	-0.089	Bad
0.98	-0.13	0.019	Good	1.42	0.13	-0.111	Bad
0.76	-0.1	0.018	Good	1.18	0.15	0.089	Bad
0.75	-0.1	0.019	Good	1.06	0.14	-0.019	Bad

0.82	-0.12	0.018	Good	0.79	0.14	0.051	Bad
0.78	-0.12	0.019	Good	0.85	0.14	-0.012	Bad
0.69	-0.09	0.019	Good	1.36	0.14	0.075	Bad
0.53	0.01	0.016	Good	0.83	0.16	0.145	Bad
0.7	-0.01	0.017	Good	1.41	0.1	0.02	Bad
0.66	0.02	0.017	Good	0.95	0.11	-0.112	Bad
0.75	0.01	0.017	Good	1.61	0.11	-0.13	Bad
0.76	-0.02	0.017	Good	0.7	0.11	0.013	Bad
0.61	0	0.017	Good	0.77	0.11	0.112	Bad
0.61	0	0.017	Good	1.15	0.15	-0.134	Bad
0.68	-0.01	0.017	Good	0.99	0.14	0.081	Bad
0.57	0	0.017	Good				
0.72	0	0.017	Good				
0.68	-0.01	0.017	Good				
0.58	0	0.016	Good				
0.59	-0.01	0.017	Good				
0.66	-0.01	0.017	Good				
1.17	-0.04	0.015	Good				
1.23	-0.07	0.018	Good				
0.9	-0.04	0.016	Good				
1.26	0.02	0.019	Good				
1.24	-0.03	0.023	Good				
1.33	-0.05	0.025	Good				
0.99	-0.06	0.026	Good				
0.85	-0.05	0.023	Good				
1.28	-0.08	0.025	Good				
0.73	-0.04	0.022	Good				
0.92	-0.03	0.025	Good				
1.07	-0.05	0.026	Good				
1.16	-0.07	0.025	Good				
1	0.05	0.026	Good				
1.22	-0.04	0.024	Good				
1.13	-0.02	0.023	Good				
0.95	-0.08	0.024	Good				
1.04	-0.06	0.025	Good				
1.2	-0.1	0.023	Good				
1.13	-0.08	0.024	Good				
1.23	-0.09	0.023	Good				

1.13	-0.09	0.024	Good
1.21	-0.04	0.023	Good
1.09	-0.04	0.024	Good

Table 2: Data of the mean and standard deviation for volume and sphericity features per fraction for good and bad response group.

	**relative change in volume**				**relative change in sphericity**			
	Good response		**Bad response**		Good response		**Bad response**	
**Fraction**	mean	Std	**mean**	**Std**	mean	Std	**mean**	**Std**
1	0	0	**0**	**0**	0	0	**0**	**0**
2	-0.109	0.228	**-0.051**	**0.198**	-0.034	0.092	**-0.021**	**0.061**
3	-0.030	0.168	**-0.056**	**0.183**	-0.013	0.060	**-0.023**	**0.066**
4	-0.137	0.185	**-0.034**	**0.210**	-0.048	0.071	**-0.016**	**0.072**
5	-0.060	0.211	**0.023**	**0.278**	-0.014	0.075	**0.000**	**0.092**
6	-0.078	0.216	**-0.092**	**0.250**	-0.028	0.076	**-0.039**	**0.089**
7	-0.165	0.237	**-0.074**	**0.202**	-0.059	0.091	**-0.030**	**0.069**
8	-0.161	0.207	**0.023**	**0.195**	-0.052	0.078	**0.004**	**0.064**
9	-0.041	0.253	**0.018**	**0.315**	-0.015	0.097	**-0.003**	**0.098**
10	-0.204	0.212	**0.004**	**0.345**	-0.075	0.092	**-0.012**	**0.122**
11	-0.083	0.179	**-0.104**	**0.239**	-0.028	0.070	**-0.042**	**0.086**
12	-0.136	0.247	**-0.097**	**0.382**	-0.043	0.094	**-0.049**	**0.129**
13	-0.226	0.181	**-0.058**	**0.349**	-0.090	0.081	**-0.032**	**0.120**
14	-0.174	0.317	**-0.059**	**0.360**	-0.062	0.122	**-0.033**	**0.123**
15	-0.119	0.318	**-0.099**	**0.248**	-0.042	0.125	**-0.041**	**0.088**

16	-0.216	0.255	**-0.157**	**0.168**	-0.081	0.102	**-0.059**	**0.061**
17	-0.312	0.141	**-0.168**	**0.153**	-0.113	0.063	**-0.062**	**0.058**
18	-0.171	0.219	**-0.039**	**0.238**	-0.046	0.077	**-0.018**	**0.077**
19	-0.169	0.219	**-0.119**	**0.256**	-0.058	0.087	**-0.049**	**0.094**
20	-0.129	0.289	**-0.173**	**0.283**	-0.045	0.108	**-0.076**	**0.130**
21	-0.214	0.201	**-0.185**	**0.231**	-0.075	0.083	**-0.072**	**0.083**
22	-0.183	0.206	**-0.135**	**0.338**	-0.073	0.082	**-0.060**	**0.117**
23	-0.136	0.206	**-0.155**	**0.187**	-0.046	0.077	**-0.058**	**0.067**
24	-0.127	0.232	**-0.016**	**0.448**	-0.046	0.092	**-0.022**	**0.135**
25	-0.243	0.201	**-0.073**	**0.332**	-0.095	0.096	**-0.018**	**0.111**
26	-0.173	0.222	**-0.167**	**0.124**	-0.075	0.098	**-0.054**	**0.044**
27	-0.008	0.220	**-0.220**	**0.160**	-0.085	0.097	**-0.036**	**0.077**
28	-0.071	0.098	**-0.281**	**0.010**	-0.080	0.097	**-0.045**	**0.061**

Supplementary Figures

Shape features didn't show significant differences between the two-response group until the end of the treatment, which suggest that these features are not very useful for early prediction of treatment response. Figures 1 shows the average relative change between the good and bad response group for the relative volume and sphericity changes showing overlap between the two-response group.

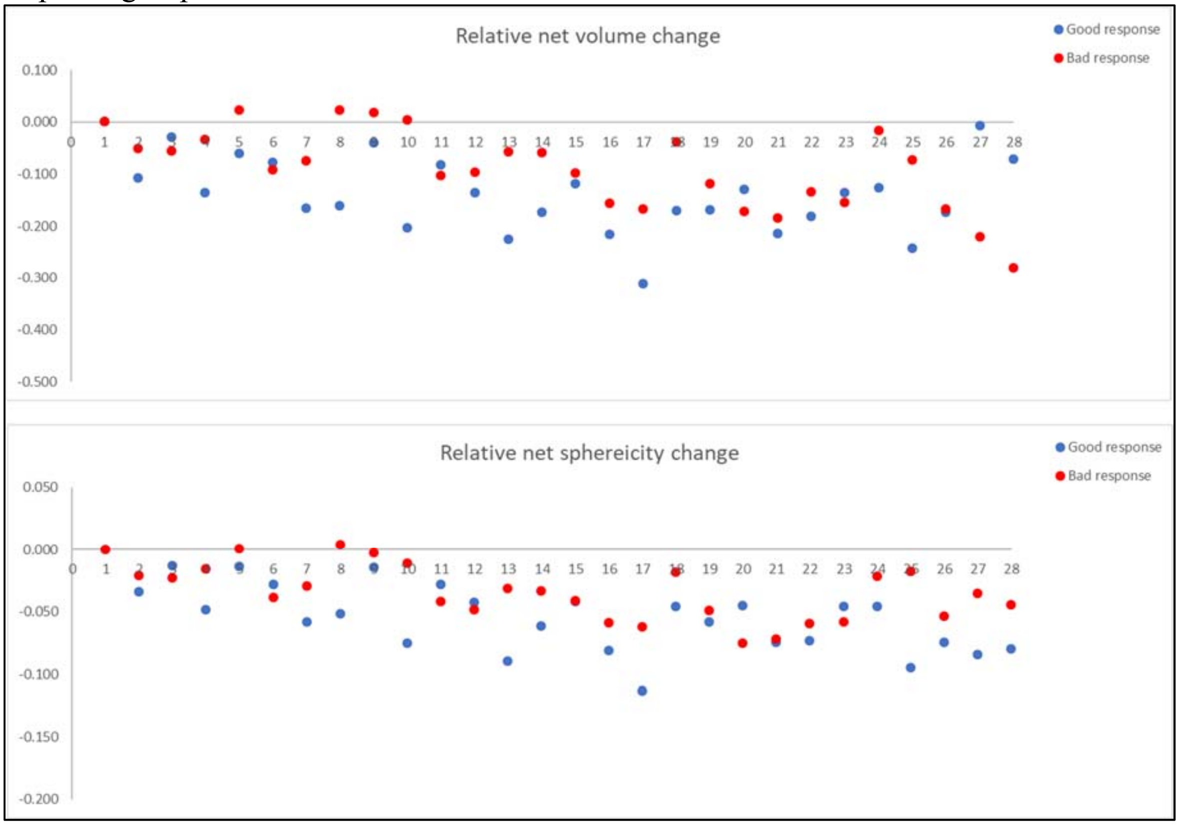

Figure 1: Average relative change per fraction for all good and bad response group for volume and sphericity changes

Figure 2 shows example boxplots of features showing significant differences between the two-response group (Mean, IDN, Entropy, and NESTD) combining all fractions and all patients in each response group.

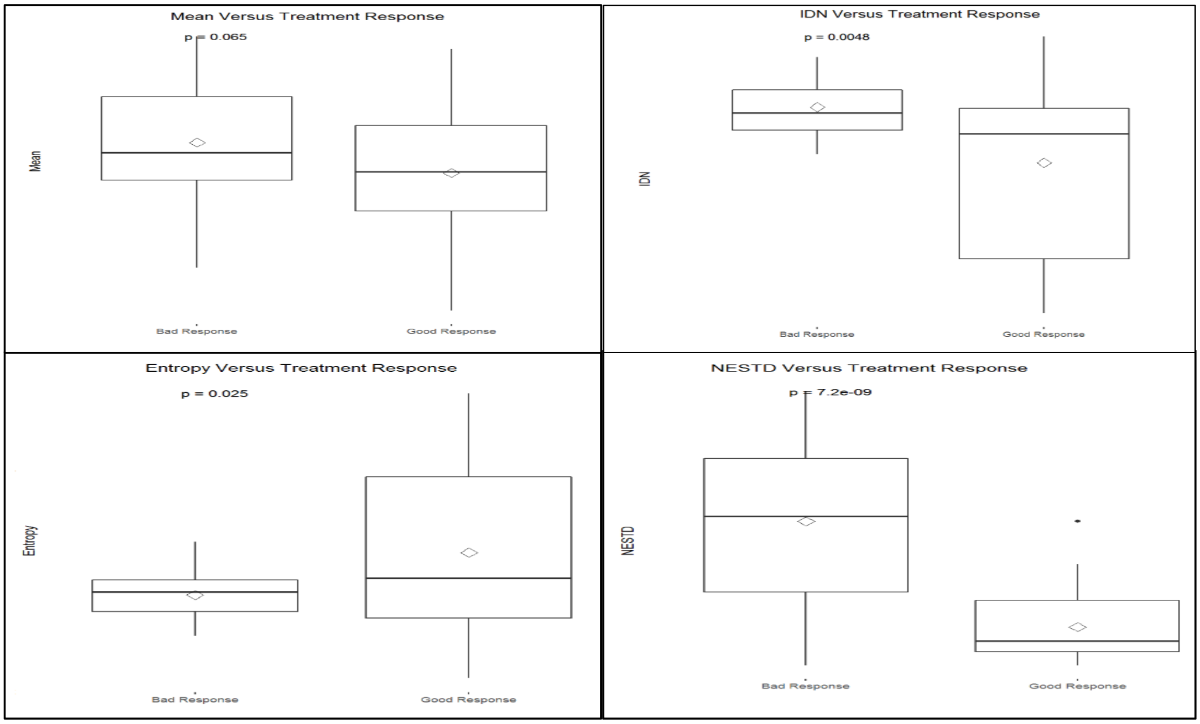

Figure 2: Examples boxplots with median and interquartile ranges for features showing significant differences between the two-response group (Mean, IDN, Entropy, and NESTD). The diamond shape represents the mean of each response group.

Figure 3 shows example boxplots of weekly change of a feature showing significant difference (Kurtosis) and a feature not showing significant difference (IQR) for all patient and all fractions in the good and bad response group

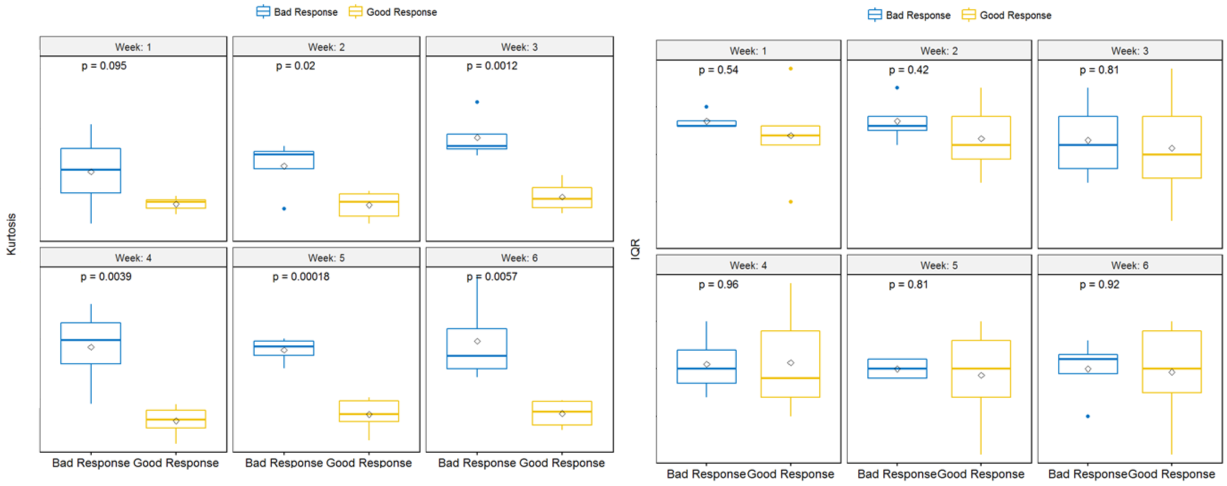

Figure 3: Example of the weekly changes for a feature showing significant difference (Kurtosis) and a feature not showing significant difference (IQR)

Figure 4 shows the confusion matrix of the confusion matrix for the training and the external independent validation sets using kurtosis, coarseness and NESTD features combination.

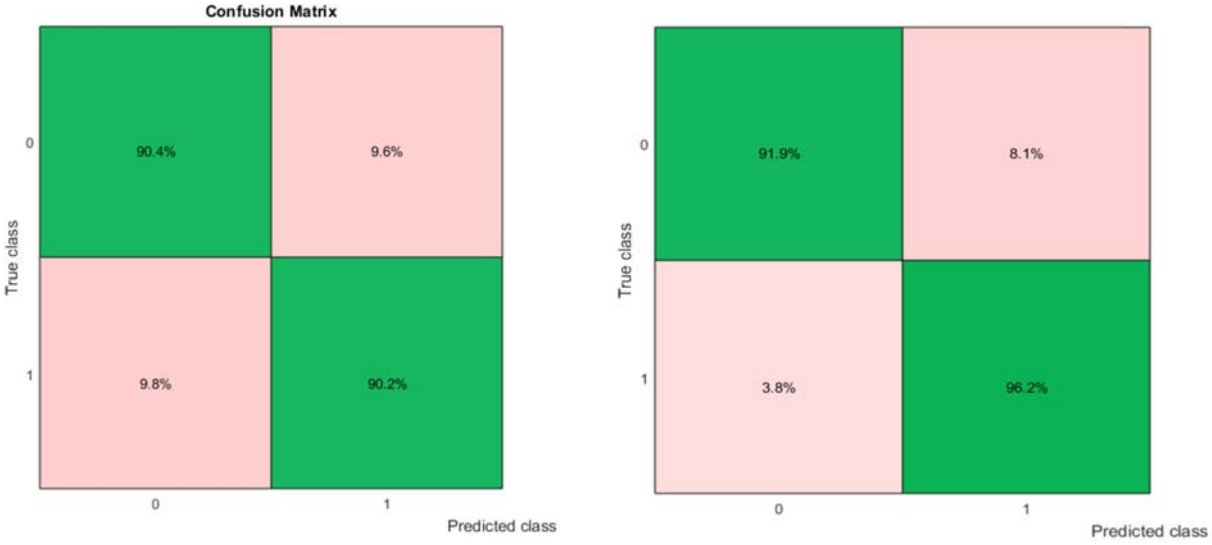

Figure 4: Right shows a confusion matrix for training set, and left shows confusion matrix of external independent validation set.
